# Dentists in Cincinnati

**Published:** 1848-07

**Authors:** 


### DENTISTS IN CINCINNATI.

BY DR. ALLEN.

When the writer of this article first commenced his professional career in Cincinnati, there were but two other dentists in the city, and these were men possessing all the necessary qualifications for eminent and skillful operators, and they still enjoy a professional reputation rarely surpassed; one of whom for many years past has been a resident in the city of Louisville, where he found a more lucrative field for his labors than in this city.

Cincinnati then contained a population of thirty thousand. Eighteen years have since elapsed, and we now have a population of some ninety thousand, showing an increase of two hundred per cent. in eighteen years. Thirty thousand of this population are foreigners, who never patronise a dentist. But this increase of population bears but a very small proportion to the increase of dentists in the city during that time. The former ratio would now give us nine dentists, instead of which we have forty-two names bearing that title, besides students and transient dentists, of whom there are always more or less in the city.

There has been a corresponding increase of dentists, we believe, throughout the country, and yet there is not one half the business done that ought to be. There are thousands of teeth lost that might be saved, causing a great amount of human suffering, that might be prevented by skilful operators, thereby converting many a sleepless night into a season of sweet repose. So great is the amount of dental services required by the community, that many laborers have been called into the field, and very many of them, it is true, without the necessary qualifications. But this should not be wondered at when we reflect that adequate facilities for acquiring them have not been within their reach. It cannot be expected that a business requiring such a wide range of theoretical and practical knowledge as that of dental surgery in all its branches, can be perfected without the requisite facilities. There is probably no other business that comprises so many different branches as that of a well educated dentist; he should understand the human system well, which is a whole profession of itself, and yet, is only a small part of ours; he should be a good mineralogtst, and a good chemist, a good porcelain manufacturer, a good moulder and caster, a good goldsmith, and a good man: these, together with several years practice, make up the sum total of a good dentist; and he who stops short of all of these acquisitions, will soon find to his mortification, that when weighed in the balance, he will be found wanting. The time was when these qualifications were not considered requisite, but that time has gone by; the dark clouds that have so long obscured the dental horizon are passing off, the thick mists of ignorance and bigotry are vanishing away, and the sun of science begins to beam upon our pathway that points to honor and to fame. We have now two institutions in our country—one in the east, the other in the west, with facilities fully commensurate to the full development of all the fundamental principles upon which our profession is based.

